# Missense variants pathogenicity annotation from homologous proteins

**DOI:** 10.1093/bioinformatics/btaf305

**Published:** 2025-05-14

**Authors:** Gabriel Ruiz-Alías, Sergi Soldevila, Xavier Altafaj, Arnau Cordomí, Mireia Olivella

**Affiliations:** Department of Biosciences, Faculty of Sciences and Technology, University of Vic-Central University of Catalonia, Vic, Barcelona 08500, Spain; Institute for Research and Innovation in Life and Health Sciences (IRIS-CC), University of Vic-Central University of Catalonia, Vic, Barcelona 08500, Spain; Department of Biosciences, Faculty of Sciences and Technology, University of Vic-Central University of Catalonia, Vic, Barcelona 08500, Spain; Institute for Research and Innovation in Life and Health Sciences (IRIS-CC), University of Vic-Central University of Catalonia, Vic, Barcelona 08500, Spain; Department of Biomedicine, School of Medicine and Health Sciences, Institute of Neurosciences, University of Barcelona, Barcelona 08036, Spain; Agustí Pi i Sunyer Biomedical Research Institute (IDIBAPS), University of Barcelona, Barcelona 08036, Spain; Department of Biochemistry and Molecular Biology, Faculty of Biosciences, Universitat Autònoma de Barcelona (UAB), Barcelona 08193, Spain; Department of Biosciences, Faculty of Sciences and Technology, University of Vic-Central University of Catalonia, Vic, Barcelona 08500, Spain; Institute for Research and Innovation in Life and Health Sciences (IRIS-CC), University of Vic-Central University of Catalonia, Vic, Barcelona 08500, Spain

## Abstract

**Motivation:**

High-throughput DNA sequencing has revealed millions of single nucleotide variants (SNVs) in the human genome, with a small fraction linked to disease. The effect of missense variants, which alter the protein sequence, is particularly challenging to interpret due to the scarcity of clinical annotations and experimental information. While using conservation and structural information, current prediction tools still struggle to predict variant pathogenicity. In this study, we explored the pathogenicity of homologous missense variants—variants in equivalent positions across homologous proteins—focusing on proteins involved in autosomal dominant diseases.

**Results:**

Our analysis of 2976 pathogenic and 17 555 non-pathogenic homologous variants demonstrated that pathogenicity can be extrapolated with 95% accuracy within a family, or up to 98% for closer homologs. Remarkably, the evaluation of 27 commonly used mutation predictor methods revealed that they were not fully capturing this biological feature. To facilitate the exploration of homologous variants, we created HomolVar, a web server that computationally predicts the pathogenesis of missense variants using annotations from homologous variants, freely available at https://rarevariants.org/HomolVar. Overall, these findings and the accompanying tool offer a robust method for predicting the pathogenicity of unannotated variants, enhancing genotype-phenotype correlations, and contributing to diagnosing rare genetic disorders.

**Availability and implementation:**

HomolVar is freely available at https://rarevariants.org/HomolVar.

## Graphical Abstract

**Figure:**
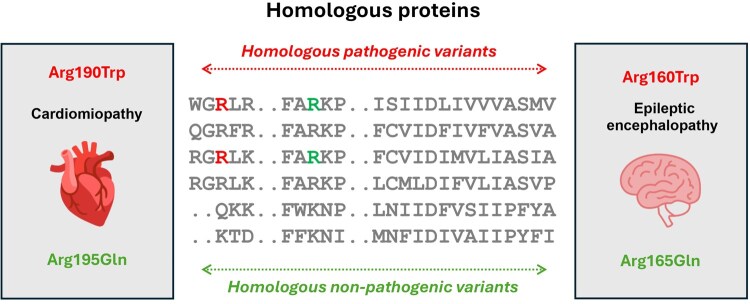

## 1 Introduction

Over the last few years, high-throughput DNA sequencing technologies have allowed the retrieval of hundreds of thousands of human genome and exome sequences, shedding light on individual genetic variation. A typical individual differs at 4.1–5.0 million sites compared to the reference human genome (Auton *et al.* 2015). Single nucleotide variations (SNV) and short indels constitute 99.9% of these variations (Gudmundsson *et al.* 2022). While most SNVs occur in non-coding regions or do not alter the amino acid sequence, missense SNVs modify the protein sequence. Consequently, missense variants can affect protein structure and function, eventually leading to disease.

Of the 4 million missense variants identified in the human genome, only 2% have a clinical annotation (pathogenic or non-pathogenic) (Cheng *et al.* 2023). In an individual’s exome, there are, on average, 200 very rare missense variants, for which it is unlikely to have clinical annotations. This includes 14–40 novel missense variants absent in other human exomes (Gudmundsson *et al.* 2022). Discerning the variants responsible for a specific condition amidst the benign variation is extremely challenging, limiting the diagnosis and therapeutic intervention in rare diseases.

For missense variants in proteins involved in autosomal dominant diseases, there is a clear correlation between the effect of a single variant in the protein structure, the protein function, and the clinical phenotype (pathogenic or non-pathogenic). Thus, numerous efforts are being made to detect the consequences of missense variants by assessing their potential to modify the structure and function of the protein. These predictions are mainly based on the phylogenetic conservation of the region containing the variant and how changes may affect protein structure and function. The first popular algorithms in this regard were SIFT (Vaser *et al.* 2016) and PROVEAN (Choi and Chan, 2015), which have been standard bearers in predicting the potential impact of variants. Other tools, such as PolyPhen-2 (Adzhubei *et al.* 2013), incorporate structural parameters for variant classification. M-CAP (Jagadeesh *et al.* 2016) and, more recently, AlphaMissense (Tordai *et al.* 2024) added machine learning-based approaches. Despite these tools having improved our knowledge about variant pathogenicity, they still have many limitations, highlighting the hard nature of this task.

In the context of GRIN-related disorders, we have recently demonstrated that homologous missense variants for proteins encoded by *GRIN* genes have identical clinical annotations (Santos-Gómez *et al.* 2022). Homologous variant extrapolation within this family of proteins, involved in a rare Mendelian disease, allowed duplicating the number of functionally and clinically annotated variants.

In the present study, we scaled up our previous analysis to all human proteins involved in autosomal dominant diseases. We show that in 95%–98% of the homologous pairs of variants that we could identify, they coincided in their annotations. Therefore, our findings demonstrate that pathogenicity can be safely extrapolated between homologous variants. Our results represent an important expansion of genetic variants’ annotation repertoire, providing a direct clinical impact on genotype-phenotype assessment in patients with rare genetic disorders.

## 2 Materials and methods

### 2.1 Build-up of pathogenic and non-pathogenic missense variant datasets

We took all reviewed human proteins in the UniProt (The UniProt Consortium 2023) as of 06 April 2023 (20 422 proteins) and exclusively kept those encoded by genes with an autosomal dominant (AD) inheritance pattern, according to OMIM (Amberger *et al.* 2019). From these, we selected only pathogenic proteins, defined as proteins with at least three reported pathogenic missense variants in ClinVar (Landrum *et al.* 2020). The final set resulted in 1282 pathogenic proteins (see Supplementary Table S1).

To construct the pathogenic missense variants dataset, all non-somatic missense disease-causing/pathogenic and likely pathogenic variants from ClinVar (06 April 2023) were collected for each protein. To create the non-pathogenic missense variants dataset, we retrieved all missense variants from gnomAD v4 (Chen *et al.* 2024) for the same group of proteins. To balance the number of pathogenic and non-pathogenic variants and to minimize the possibility of taking pathogenic rare variants yet to be identified, we generated a collection of datasets discarding variants with allele frequencies up to various thresholds (see Supplementary Table S2). Based on these results, the final analysis was performed by discarding gnomAD variants with an allele frequency below 10^−6^. In addition, we discarded variants with unclear consequences and those labelled as pathogenic in ClinVar and present also in gnomAD (assumed non-pathogenic). The latter are probably neutral variants that were wrongly identified as disease-causing in patients with a disorder due to the lack of these variants in the healthy population. Still, we opted to discard them provided that the non-pathogenic set was large enough. The obtained datasets contained 28 888 pathogenic missense variants (see Supplementary Table S3) and 310 268 non-pathogenic missense variants (see Supplementary Table S4), all involving pathogenic proteins (see above).

### 2.2 Identification of homologous variants in pathogenic and non-pathogenic missense variant datasets

We used Pfam (Paysan-Lafosse *et al.* 2023) family multiple sequence alignments to identify equivalent positions between homologous proteins to those present in our previously described datasets of missense variants in pathogenic proteins. In particular, manually curated *seed* alignments (Sonnhammer *et al.* 1998) were selected instead of non-manually curated *full* alignments. We considered two different criteria for homologous variants: (i) those that imply the same amino acid change at the same equivalent position (*strict pairs*), or (ii) those involving similar amino acid change (positive score in the BLOSUM62 substitution matrix, see Supplementary Table S5) at the same equivalent position (*similar pairs*). The BLOSUM62 was chosen as the average sequence similarity between the proteins containing homologous variants was around 60%. To discard the possible influence of the choice of the substitution matrix, we repeated the analysis using BLOSUM45 and BLOSUM80 matrices as well.

### 2.3 Web server

The HomolVar web application was constructed using a Python backend (v.3.10.12) with the Flask framework (v.3.0.3). The application was deployed using Apache v.2.4.52.

### 2.4 Comparison of HomolVar to other mutation predictors

For comparison purposes, the pathogenicity of all pairs of homologous variants was predicted using dbNSFP (Liu *et al.* 2016), which includes SIFT (Vaser *et al.* 2016), PolyPhen-2 (Adzhubei *et al.* 2013), AlphaMissense (Tordai *et al.* 2024), MutationTaster (Schwarz *et al.* 2014), MutationAssessor (Reva *et al.* 2011), PROVEAN (Choi and Chan, 2015), VEST (Carter *et al.* 2013), M-CAP (Jagadeesh *et al.* 2016), REVEL (Ioannidis *et al.* 2016), MVP (Qi *et al.* 2018), gMVP (Zhang *et al.* 2022), PrimateAI (Sundaram *et al.* 2018), deogen2 (Raimondi *et al.* 2017), ClinPred (Alirezaie *et al.* 2018), LIST-S2 (Malhis *et al.* 2020), ESM1b (Brandes *et al.* 2023), MutScore (Quinodoz *et al.* 2022), CADD (Kircher *et al.* 2014), DANN (Quang *et al.* 2015), fathmm-XF (Rogers *et al.* 2018), BayesDel (Feng, 2017), MetaSVM (Kim *et al.* 2017), MetaLR (Chen *et al.* 2023), MetaRNN (Li *et al.* 2022), VARITY (Wu *et al.* 2021), phyloP (Pollard *et al.* 2010), and phastCons (Siepel *et al.* 2005).

## 3 Results

### 3.1 Comparing pathogenicity on pairs of homologous variants

In this study, we explored the hypothesis that similar variants affecting equivalent positions of homologous proteins, the so-called “homologous variants”, result in conserved pathogenicity. We focused the analysis on “pathogenic proteins,” defined as proteins linked to autosomal dominant diseases. In these proteins, there is a strong correlation between the effect of a single missense variant in the protein structure and function, as well as with the clinical outcome (pathogenic or non-pathogenic). Thus, this group excludes proteins fulfilling one or more of the following criteria: (i) not primarily related to human diseases, (ii) encoded by genes with recessive inheritance, and/or (iii) linked to complex diseases, a scenario that would mitigate the interpretation of the functional effect of the genetic variant.

Methodologically, we used family alignments to map equivalent positions within homologous proteins and publicly available missense variant data [such as ClinVar (Landrum *et al.* 2020) and gnomAD (Chen *et al.* 2024)] to test our hypothesis. Due to sequence conservation heterogeneity, only those variants located within conserved regions (i.e. available sequence alignments with the corresponding homologous proteins) were analyzed. This resulted in a collection of 2976 pathogenic and 17 555 non-pathogenic variants distributed along 1282 proteins and 292 Pfam family domains (see Methods) (see Supplementary Table S6).

We first analyzed variants in equivalent positions with the same amino acid change (named “*strict pairs*,” Table 1). We found 2482 (95%) coincident pairs of homologous variants, that is, presenting the same annotation [1612 with both variants annotated as non-pathogenic (N-N), and 870 variants annotated as pathogenic (P-P)]. Only 127 homologous pairs presented discordant annotations (N-P; one non-pathogenic and one pathogenic) (see Table 1 and Supplementary Table S7). The computed classification evaluation metrics [sensitivity, specificity, accuracy, and Matthews correlation coefficient (MCC)] showed all high values, near 1. These data strongly supported the hypothesis that the pathogenicity of variants lacking annotations can be inferred from annotated variants in homologous positions with high predictive power. Moreover, as the quality of multiple sequence alignments increases, the number of annotations that can be extrapolated between homologous variants will also increase. In terms of specificity, the results are in agreement with a preprint manuscript that used a similar approach on paralogous variants using data exclusively from ClinVar (Li *et al.* 2023). In that work, however, they obtained a very low sensitivity, probably due to the lack of a large dataset of non-pathogenic variants –such as the one employed in the present study-, and an appropriate distinction between variants in pathogenic proteins from those on non-pathogenic proteins.

**Table 1.** Comparison of genetic variants pathogenicity between pairs of homologous variants.^a^

		N-N (TN)	P-P (TP)	N-P (FN+FP)	Total pairs	Sensitivity	Specificity	Accuracy	MCC
Strict pairs	All	1612	870	127	2609	0.93	0.96	0.95	0.89
	>30% SI	632	823	30	1485	0.98	0.98	0.98	0.96
Similar mutated aa	All	2164	1433	178	3775	0.94	0.96	0.95	0.90
	>30% SI	909	1376	60	2345	0.98	0.97	0.97	0.95
Similar reference aa	All	1650	872	150	2672	0.92	0.96	0.94	0.88
	>30% SI	556	824	33	1413	0.98	0.97	0.98	0.95
Similar reference and mutated aa	All	3074	1444	266	4784	0.92	0.96	0.94	0.87
	>30%SI	1083	1381	89	2553	0.97	0.96	0.97	0.93

a Comparison of genetic variants pathogenicity between pairs of homologous variants, including strict pairs (same amino acids) and similar pairs (amino acids with similar physicochemical properties). The analysis was conducted for the group of homologous variant pairs (“All”) and proteins that share more than 30% sequence identity (>30% SI) to weed out proteins that are distantly related. SI, sequence identity; aa, amino acids; N-N, non-pathogenic variants; P-P, pathogenic variants; N-P, discordant (non-pathogenic/pathogenic) variants; MCC, Matthew’s correlation coefficient.

It is reasonable to speculate that discrepancies in non-coincident pairs may be due to distantly related homologous proteins or to the misalignment of homologous sequences that also occur at lower sequence identities. To verify this hypothesis, we discarded those protein pairs with <30% sequence identity in their aligned domains. The subsequent analysis revealed that the number of homologous variant pairs with identical pathogenicity increased to 98% (Table 1), although with the penalty of decreasing the number of pairs. These results showed that high protein conservation is directly linked with the reliability of annotation extrapolation. Moreover, these results suggest that developing robust sequence alignments within a protein family curated by experts and eventually incorporating structural information would improve the performance of pathogenesis extrapolation.

Despite the high number of missense variants identified in the human genome, those with an associated clinical annotation are still scarce. Therefore, to increase the number of annotations through homologous variant extrapolation, we checked whether variants could also be extrapolated, not only for the same variation but also for similar ones, that is, amino acids with similar physicochemical properties. We compared pairs of homologous variants presenting a similar reference and/or a similar mutated amino acid according to BLOSUM62 scores, consistent with an average sequence similarity between protein pairs of 60%. Two amino acids are considered similar when their substitution score in the matrix is >0. For instance, Leu would be considered similar to Ile (score = 2), Met (score = 2) and Val (score = 1) (see Supplementary Table S5).

The results showed that by incorporating similar mutated amino acid changes, the dataset increased while not affecting the classification evaluation metrics (see Table 1 and Supplementary Table S8). In contrast, by incorporating similar reference amino acids, the number of pairs remained almost the same as for strict pairs, and the statistical descriptors were slightly reduced (see Table 1 and Supplementary Table S9). This might be explained by the fact that the effect of a missense variant is more dependent on the physicochemical properties of the mutated amino acid rather than the reference amino acid. The advantage of incorporating similar reference and mutated amino acids is that it almost duplicates the dataset size without compromising the comparison evaluation metrics (see Table 1 and Supplementary Table S10). We repeated the same analysis using BLOSUM45 and BLOSUM80 substitution matrices to evaluate the possible effect of the substitution matrix on evaluating similarity. These matrices are appropriate for proteins with lower or higher sequence identity, respectively. The results confirmed that the choice of matrix did not notably impact the outcomes (see Supplementary Table S11).

We asked ourselves if domains could also be classified as pathogenic and non-pathogenic or if their pathogenesis was related to the pathogenicity of the protein. It is estimated that 70% of all human proteins present more than one domain (Sonnhammer *et al.* 1998). For all domains containing disease-causing variants, we assessed if the same domain was also present in non-pathogenic proteins. Supplementary Table S12 shows that these domains are either present in pathogenic and non-pathogenic proteins, suggesting that the pathogenicity is not associated with specific domains and that it is associated with specific proteins. Thus, if a variant affects the function of a domain, this variant will be disease-causing if the domain is contained in pathogenic proteins (proteins involved in autosomal dominant inheritance disorders) but will be neutral if the domain is contained in proteins not involved in any disease, involved only in complex diseases or follow an X-linked or recessive inheritance.

### 3.2 Homologous variant example: Ion transport family domain

**Figure 1.**
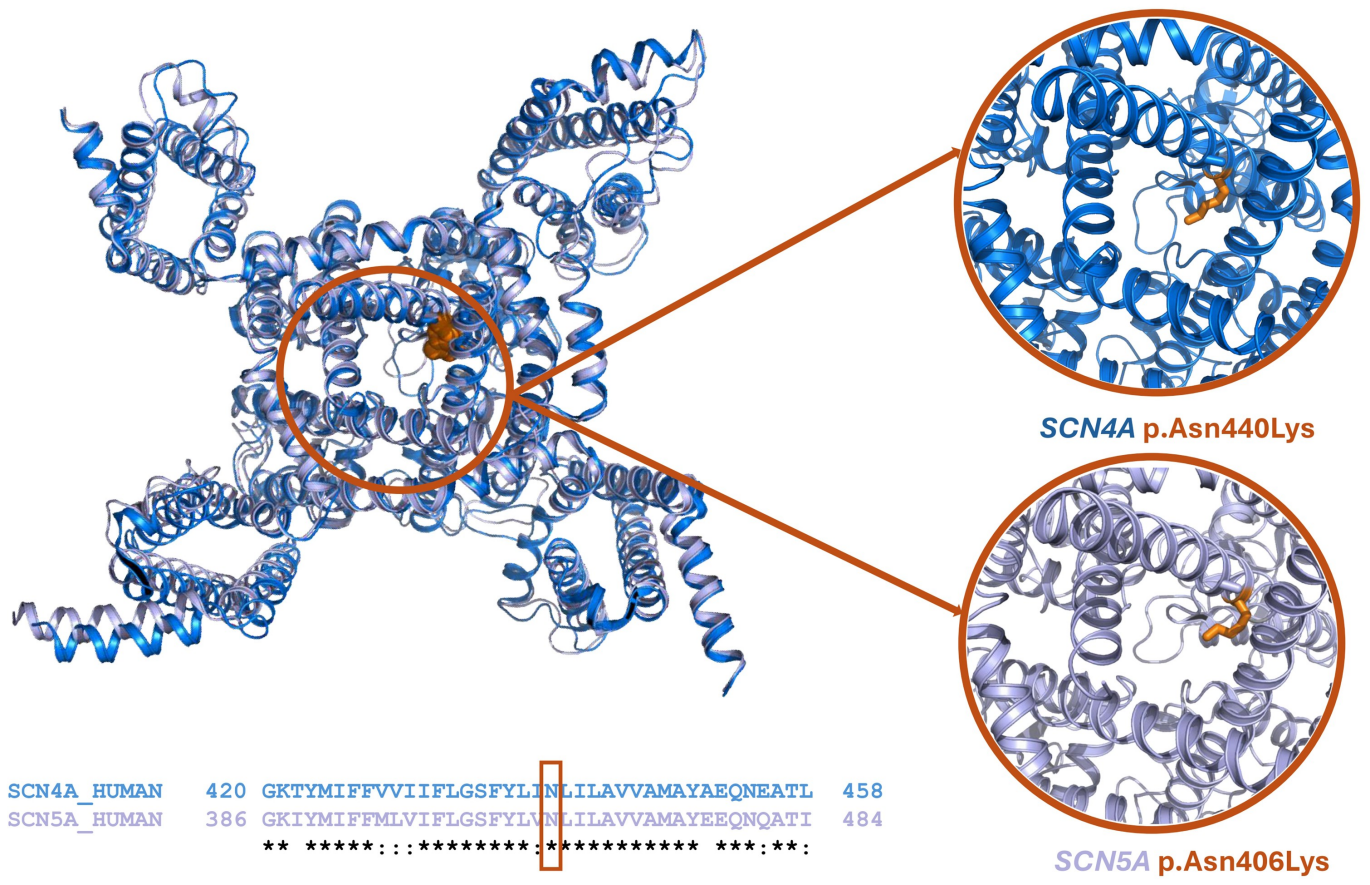
Top-left: structural superimposition of *SCN4A* [in dark blue; PDB ID: 6AGF (Pan *et al.* 2018)] and *SCN5A* [in purple; PDB ID: 6LQA (Li *et al.* 2021)], showing Asn440 and Asn406 as orange spheres. Bottom-left: sequence alignment of the Ion Channel domain for SCN4A and SCN5A. Right: Variants p.Asn440Lys in *SCN4A* and p.Asn410Lys in *SCN5A* (as orange sticks) are pointing toward the center of the pore channel.

To illustrate the basis of the pathogenicity extrapolation between homologous variants, we thoroughly characterized discrete missense mutations affecting the Ion Transport protein family domain (PF00520). Although this domain is present in 102 human proteins, curated multiple sequence alignments (seed Pfams) are only available for 14 pathogenic and 9 non-pathogenic proteins. For one of its members, SCN4A, variant p. Asn440Lys has been associated with paramyotonia congenita, a channelopathy altering muscle contraction (Lehmann-Horn *et al.* 2011, Lossin *et al.* 2012). The structure of this channel (Fig. 1) shows that residue Asn440 points towards the center of the pore channel. This orientation suggests that the positive charge introduced by mutation p. Asn440Lys would modify channel gating, altering Na+ influx and ultimately affecting membrane potential. Figure 1 displays the sequence alignment between SCN4A-encoded protein and its paralogous SCN5A gene product, sharing 66% sequence identity. The structure superimposition of the two sodium channels is almost fully coincident, with a root mean squared standard deviation of 1.0 Å. The equivalent position of SCN4A(p.Asn440) corresponds to SCN5A(p.Asn406), and is located within a conserved region. Consequently, the residues appear in the same position, at the center of the ion channel pore, and might have similar functional outcomes, namely a disturbance of channel gating properties. In line with the conserved topology and structural-based prediction, SCN5A(p.Asn406Lys) variant has also been reported to be disease-associated, causing a cardiac channelopathy (Tester *et al.* 2005). The same homologous variant is also found, annotated as pathogenic, in SCN1A, SCN9A, and CAC1A. According to our results, the pathogenicity of these variants can be extrapolated to predict as pathogenic 43 potential variants that mutate to Lys in the same equivalent position in the PF000520 Multiple Sequence Alignment, in the 14 proteins that are associated with autosomal dominant inheritance. Furthermore, if we extend this prediction to variants that result in similar amino acids, an additional 132 variants across these 14 proteins can be predicted as pathogenic. Consequently, one single variant, SCN4A(p.Asn440Lys) allows us to predict the pathogenesis of 14 identical homologous potential variants and, in addition, 132 similar homologous potential variants (see Supplementary Table S13).

On the other hand, three identical homologous variants are present in the same position of the alignment for seven additional members of the Transport protein family domain that do not follow an autosomal dominant inheritance (see Supplementary Table S13). These variants are present in gnomAD and are classified as non-disease-causing. Although these variants may affect the structure and/or the function of the proteins, these proteins are associated with autosomal recessive, X-linked inheritance, only affected by copy number variations or not related to any disease. In accordance with our results, pathogenic homologous variants can also be used to identify homologous variants in proteins not related to an autosomal dominant inheritance that are affecting the structure and/or function of the protein, but that are not disease-causing per se and are found in healthy population.

### 3.3 HomolVar web server

**Figure 2.**
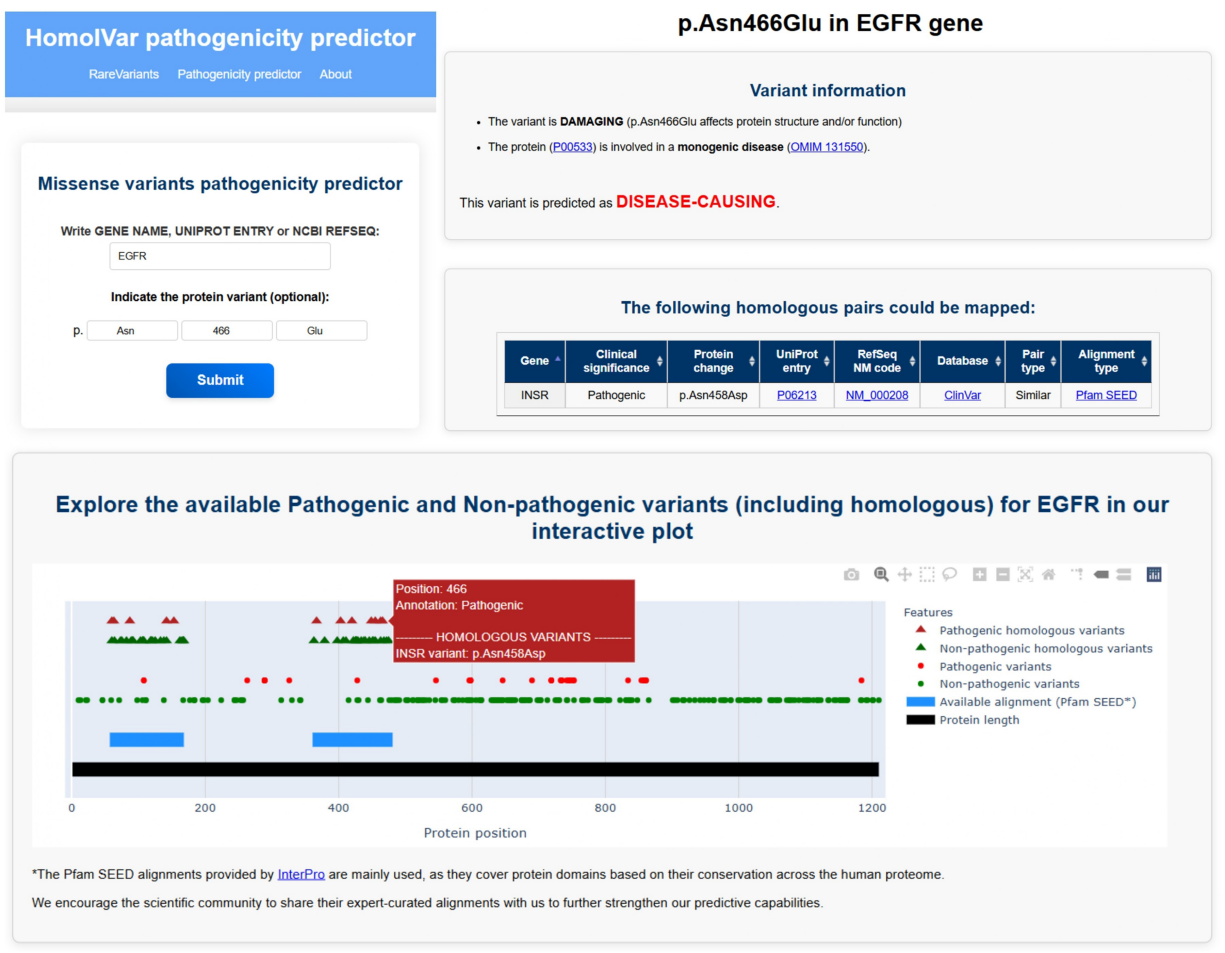
Overview of the HomolVar interface and functionality. (top-left) Input panel where users can specify a protein and optionally a specific variant. (top-right and bottom) mosaic of output panels consisting of prediction, the list of mapped homologous pairs and an interactive plot displaying the variants already annotated for the protein and the homologous variants.

To facilitate the task of encountering homologous variants and their annotations, we developed a web server that computationally predicts the pathogenesis of missense variants based on available pathogenesis annotations of homologous variants. HomolVar aligns the query variant provided by the user to the corresponding multiple sequence alignments to identify homologous variants (either identical or similar) with available annotations. These are used to classify the variants as *damaging* or *not damaging* the protein structure and function. If the variant is predicted as *damaging* the protein structure and function and the protein is involved in a monogenic disease, the variant is predicted as *disease-causing*. If the protein is not involved in a monogenic disease, then the variant is predicted as *non-disease-causing* because, even though it may alter protein structure and function, this is not enough to cause pathogenicity. For each query (see Fig. 2 for an example), which may be a specific variant or the whole gene/protein, the output displays the predictions together with all available annotations for homologous variants. A full protein schematic interactive representation of all the annotated variants and homologous variants along the sequence is also displayed.

### 3.4 Comparison of HomolVar to other mutation predictors

Next, we questioned if this biological feature of homologous variants is worth considering explicitly or if it is already captured by the commonly used algorithms for variant prediction. For this purpose, we compared HomolVar prediction power to 27 mutation predictors for homologous variants (see Table 2, Supplementary Tables S14 and 15). The successful prediction rate for mutation prediction servers ranged between 34% and 79%, approximately 54% on average. The low pathogenicity prediction rates in the mutation prediction tools show that the concept of homology within variants is not considered or properly captured by these mutation prediction servers either explicitly or implicitly. The results claim the need to incorporate homologous annotations. Yet, it should be taken into account that these results are already overestimated due to circularity problems (Grimm *et al.* 2015). Circularity is due to overlapping variants in the training and evaluation dataset and also to protein overlap when variants from the same protein appear in the training and evaluation dataset. It has been extensively discussed that mutation predictors that are based on phylogenetic trees rather than multiple sequence alignments present a better prediction power (Siepel *et al.* 2005, Pollard *et al.* 2010, Dereli *et al.* 2024). However, this is not observed in our dataset, where methods based on phylogenetic trees do not show a better performance.

**Table 2.** Predictive power of some commonly used mutation compared to HomolVar.

Mutation predictor method	Predictive power
HomolVar	0.95
gMVP	0.79
PrimateAI	0.79
AlphaMissense	0.76
SIFT	0.58
FathMM	0.53
PolyPhen-2	0.50
PhyloP17way_primate	0.43
CADD	0.38

Predictive power of some commonly used mutation predictor methods compared to HomolVar (between 0 and 1). The performance is computed as the correct number of variants pathogenicity predictions divided by the total number of variants, for all pairs of identical homologous variants.

### 3.5 Strengths and limitations of HomolVar

We have previously used homologous annotations to predict the pathogenesis of unannotated variants within specific families of proteins (Santos-Gómez *et al.* 2022). In the present study, we have extended this assumption to all proteins in the human proteome. Although it is expected that sequence similarity implies similarity in the variant label, an appropriate assessment had, to the best of our knowledge, not been performed before. HomolVar incorporates this biological feature, which is not properly captured by current mutation prediction algorithms.

An important feature we have set in HomolVar is distinguishing between variants located in pathogenic proteins or non-pathogenic proteins. That is, although a variant can affect the function of a protein (damaging variants), if the protein is not involved in an autosomal dominant inheritance disease, then the variant can be found in a healthy population, and the variant is not disease-causing. In general, this assumption is not considered in mutation predictors. Thus, the training sets mix variants in pathogenic and non-pathogenic proteins, decreasing its prediction power. The inclusion of proteins involved in autosomal recessive disorders, X-linked disorders or complex diseases implies having a dataset where there is no direct correlation between the effect of a change in the structure and function of a protein and the corresponding clinical phenotype. Moreover, we propose that mutation predictors should differentiate between predicting that a variant is affecting the structure and/or function of the protein (damaging/not damaging) and additionally, if the variant is responsible for a clinical phenotype (disease-causing/non-disease-causing).

One of the current limitations of HomolVar is the number of available annotated pathogenic missense variants and non-pathogenic missense variants in pathogenic proteins, which reduces its coverage. Another limitation that also reduces the coverage is the lack of high-quality multiple sequence alignments for all positions in all human proteins and all its members. However, due to the expected increase in genome and exome sequencing and to the availability of improved multiple sequence alignments for all human proteins, it is expected that the coverage and the prediction power of HomolVar will increase in future years. HomolVar web server is ready to incorporate both upcoming new variants and user-provided multiple sequence alignments at different levels of protein classification.

## 4 Conclusions

Our analysis demonstrates that the pathogenicity within homologous variants in pathogenic proteins is coincident within homologous variants with an accuracy of 95%. This applies to both identical homologous variants (same reference and mutated amino acids) and also to similar homologous variants (reference and mutated amino acids with similar physicochemical properties). We also show that the accuracy can reach up to 98% when imposing a minimum threshold of 30% sequence identity within the protein domain.

The present findings can be useful for extrapolating the pathogenicity of annotated variants into non-annotated homologous variants, broadening the number of annotations. Although the hypothesis we have tested is quite simple, we have observed that this information is not being considered in commonly used mutation predictors, which reach moderate prediction rates between 34 and 79%, when considering variations with available annotations in the original protein and homologous ones. Still, these values may be overestimated due to the circularity of variants in the training and test sets.

Considering the scarce number of variants with pathogenicity annotations, these findings are clinically highly relevant, with outstanding importance in the context of patient diagnosis and personalized therapies. Additionally, we expect that the concept of homologous missense variants is valid beyond the context used in the present study. In proteins involved in complex diseases or recessive disorders, the pathogenicity annotations from missense variants in pathogenic proteins could be used to understand their putative aetiological contribution. In the same direction, these extrapolations would be potentially used to annotate somatic mutations or to select a conserved variant in orthologous proteins for the design and generation of transgenic models of human genetic disorders.

We created a web server (HomolVar, available at https://rarevariants.org/HomolVar/) that facilitates this task of exploring homologous variations and their annotations.

## Supplementary Material

btaf305_Supplementary_Data

## Data Availability

The code developed for this project is available at (https://github.com/ruizzgabriel/Annotating-pathogenicity-of-missense-variants-from-homologous-proteins/tree/main), and the datasets can be accessed at (10.5281/zenodo.14415437).

## References

[btaf305-B1] Adzhubei I , Jordan DM, Sunyaev SR. Predicting functional effect of human missense mutations using PolyPhen-2. Curr Protoc Hum Genet 2013;20:1–50. 10.1002/0471142905.hg0720s76 PMC4480630 23315928

[btaf305-B2] Alirezaie N , Kernohan KD, Hartley T et al ClinPred: prediction tool to identify disease-relevant nonsynonymous single-nucleotide variants. Am J Hum Genet 2018;103:474–83. 30220433 10.1016/j.ajhg.2018.08.005 PMC6174354

[btaf305-B3] Amberger JS , Bocchini CA, Scott AF et al OMIM.org: leveraging knowledge across phenotype-gene relationships. Nucleic Acids Res 2019;47:D1038–43. 30445645 10.1093/nar/gky1151 PMC6323937

[btaf305-B4] Auton A , Brooks LD, Durbin RM et al A global reference for human genetic variation. Nature 2015;526:68–74. 26432245 10.1038/nature15393 PMC4750478

[btaf305-B5] Brandes N , Goldman G, Wang CH et al Genome-wide prediction of disease variant effects with a deep protein language model. Nat Genet 2023;55:1512–22. 37563329 10.1038/s41588-023-01465-0 PMC10484790

[btaf305-B6] Carter H , Douville C, Stenson PD et al Identifying Mendelian disease genes with the variant effect scoring tool. BMC Genomics 2013;14 Suppl 3:S3. 10.1186/1471-2164-14-S3-S3 PMC3665549 23819870

[btaf305-B7] Chen S , Francioli LC, Goodrich JK et al A genomic mutational constraint map using variation in 76,156 human genomes. Nature 2024;625:92–100. 38057664 10.1038/s41586-023-06045-0 PMC11629659

[btaf305-B8] Chen Y , Liu L, Li J, et al MetaLR: meta-tuning of learning rates for transfer learning in medical imaging. In: Greenspan H et al (ed.), Medical Image Computing and Computer Assisted Intervention—MICCAI 2023. Cham: Springer Nature Switzerland, 2023, 706–16.

[btaf305-B9] Cheng J , Novati G, Pan J et al Accurate proteome-wide missense variant effect prediction with AlphaMissense. Science 2023;381:eadg7492. 37733863 10.1126/science.adg7492

[btaf305-B10] Choi Y , Chan AP. PROVEAN web server: a tool to predict the functional effect of amino acid substitutions and indels. Bioinformatics 2015;31:2745–7. 25851949 10.1093/bioinformatics/btv195 PMC4528627

[btaf305-B11] Dereli O , Kuru N, Akkoyun E et al PHACTboost: a Phylogeny-Aware pathogenicity predictor for missense mutations via boosting. Mol Biol Evol 2024;41:msae136. 38934805 10.1093/molbev/msae136 PMC11251492

[btaf305-B12] Feng B-J. PERCH: a unified framework for disease gene prioritization. Hum Mutat 2017;38:243–51. 27995669 10.1002/humu.23158 PMC5299048

[btaf305-B13] Grimm DG , Azencott C-A, Aicheler F et al The evaluation of tools used to predict the impact of missense variants is hindered by two types of circularity. Hum Mutat 2015;36:513–23. 25684150 10.1002/humu.22768 PMC4409520

[btaf305-B14] Gudmundsson S , Singer-Berk M, Watts NA et al Variant interpretation using population databases: lessons from gnomAD. Hum Mutat 2022;43:1012–30. 34859531 10.1002/humu.24309 PMC9160216

[btaf305-B15] Ioannidis NM , Rothstein JH, Pejaver V et al REVEL: an ensemble method for predicting the pathogenicity of rare missense variants. Am J Hum Genet 2016;99:877–85. 27666373 10.1016/j.ajhg.2016.08.016 PMC5065685

[btaf305-B16] Jagadeesh KA , Wenger AM, Berger MJ et al M-CAP eliminates a majority of variants of uncertain significance in clinical exomes at high sensitivity. Nat Genet 2016;48:1581–6. 27776117 10.1038/ng.3703

[btaf305-B17] Kim S , Jhong J-H, Lee J et al Meta-analytic support vector machine for integrating multiple omics data. BioData Min 2017;10:2. 28149325 10.1186/s13040-017-0126-8 PMC5270233

[btaf305-B18] Kircher M , Witten DM, Jain P et al A general framework for estimating the relative pathogenicity of human genetic variants. Nat Genet 2014;46:310–5. 24487276 10.1038/ng.2892 PMC3992975

[btaf305-B19] Landrum MJ , Chitipiralla S, Brown GR et al ClinVar: improvements to accessing data. Nucleic Acids Res 2020;48:D835–D844. 31777943 10.1093/nar/gkz972 PMC6943040

[btaf305-B20] Lehmann-Horn F , Orth M, Kuhn M et al A novel N440K sodium channel mutation causes myotonia with exercise-induced weakness—exclusion of CLCN1 exon deletion/duplication by MLPA. Acta Myol 2011;30:133–7. 22106717 PMC3235863

[btaf305-B21] Li C , Zhi D, Wang K et al MetaRNN: differentiating rare pathogenic and rare benign missense SNVs and InDels using deep learning. Genome Med 2022;14:115. 36209109 10.1186/s13073-022-01120-z PMC9548151

[btaf305-B22] Li N , Theotokis P, Zhang X et al Variant annotation across homologous proteins (“Paralogue Annotation”) identifies disease-causing missense variants with high precision, and is widely applicable across protein families. bioRxiv 2023. 10.1101/2023.08.07.552236

[btaf305-B23] Li Z , Jin X, Wu T et al Structural basis for pore blockade of the human cardiac sodium channel nav 1.5 by the antiarrhythmic drug quinidine. Angew Chem Int Ed Engl 2021;60:11474–80. 33684260 10.1002/anie.202102196

[btaf305-B24] Liu X , Wu C, Li C et al dbNSFP v3.0: a One-Stop database of functional predictions and annotations for human nonsynonymous and Splice-Site SNVs. Hum Mutat 2016;37:235–41. 26555599 10.1002/humu.22932 PMC4752381

[btaf305-B25] Lossin C , Nam T-S, Shahangian S et al Altered fast and slow inactivation of the N440K Nav1.4 mutant in a periodic paralysis syndrome. Neurology 2012;79:1033–40. 22914841 10.1212/WNL.0b013e3182684683

[btaf305-B26] Malhis N , Jacobson M, Jones SJM et al LIST-S2: taxonomy based sorting of deleterious missense mutations across species. Nucleic Acids Res 2020;48:W154–61. 32352516 10.1093/nar/gkaa288 PMC7319545

[btaf305-B27] Pan X , Li Z, Zhou Q et al Structure of the human voltage-gated sodium channel Nav1.4 in complex with β1. Science 2018;362:eaau2486. 30190309 10.1126/science.aau2486

[btaf305-B28] Paysan-Lafosse T , Blum M, Chuguransky S et al InterPro in 2022. Nucleic Acids Res 2023;51:D418–D427. 36350672 10.1093/nar/gkac993 PMC9825450

[btaf305-B29] Pollard KS , Hubisz MJ, Rosenbloom KR et al Detection of nonneutral substitution rates on mammalian phylogenies. Genome Res 2010;20:110–21. 19858363 10.1101/gr.097857.109 PMC2798823

[btaf305-B30] Qi H , Zhang H, Zhao Y, et al MVP: predicting pathogenicity of missense variants by deep learning. Nat Commun 2018;12:510. 10.1038/s41467-020-20847-0 PMC7820281 33479230

[btaf305-B31] Quang D , Chen Y, Xie X et al DANN: a deep learning approach for annotating the pathogenicity of genetic variants. Bioinformatics 2015;31:761–3. 25338716 10.1093/bioinformatics/btu703 PMC4341060

[btaf305-B32] Quinodoz M , Peter VG, Cisarova K et al Analysis of missense variants in the human genome reveals widespread gene-specific clustering and improves prediction of pathogenicity. Am J Hum Genet 2022;109:457–70. 35120630 10.1016/j.ajhg.2022.01.006 PMC8948164

[btaf305-B33] Raimondi D , Tanyalcin I, Ferté J et al DEOGEN2: prediction and interactive visualization of single amino acid variant deleteriousness in human proteins. Nucleic Acids Res 2017;45:W201–W206. 28498993 10.1093/nar/gkx390 PMC5570203

[btaf305-B34] Reva B , Antipin Y, Sander C et al Predicting the functional impact of protein mutations: application to cancer genomics. Nucleic Acids Res 2011;39:e118. 21727090 10.1093/nar/gkr407 PMC3177186

[btaf305-B35] Rogers MF , Shihab HA, Mort M et al FATHMM-XF: accurate prediction of pathogenic point mutations via extended features. Bioinformatics 2018;34:511–3. 28968714 10.1093/bioinformatics/btx536 PMC5860356

[btaf305-B36] Santos-Gómez A , García-Recio A, Miguez-Cabello F et al Identification of homologous GluN subunits variants accelerates GRIN variants stratification. Front Cell Neurosci 2022;16:998719. 36619673 10.3389/fncel.2022.998719 PMC9816381

[btaf305-B37] Schwarz JM , Cooper DN, Schuelke M et al MutationTaster2: mutation prediction for the deep-sequencing age. Nat Methods 2014;11:361–2. 24681721 10.1038/nmeth.2890

[btaf305-B38] Siepel A , Bejerano G, Pedersen JS et al Evolutionarily conserved elements in vertebrate, insect, worm, and yeast genomes. Genome Res 2005;15:1034–50. 16024819 10.1101/gr.3715005 PMC1182216

[btaf305-B39] Sonnhammer EL , Eddy SR, Birney E et al Pfam: multiple sequence alignments and HMM-profiles of protein domains. Nucleic Acids Res 1998;26:320–2. 9399864 10.1093/nar/26.1.320 PMC147209

[btaf305-B40] Sundaram L , Gao H, Padigepati SR et al Predicting the clinical impact of human mutation with deep neural networks. Nat Genet 2018;50:1161–70. 30038395 10.1038/s41588-018-0167-z PMC6237276

[btaf305-B41] Tester DJ , Will ML, Haglund CM et al Compendium of cardiac channel mutations in 541 consecutive unrelated patients referred for long QT syndrome genetic testing. Heart Rhythm 2005;2:507–17. 15840476 10.1016/j.hrthm.2005.01.020

[btaf305-B42] The UniProt Consortium. UniProt: the universal protein knowledgebase in 2023. Nucleic Acids Res 2023;51:D523–D531. 36408920 10.1093/nar/gkac1052 PMC9825514

[btaf305-B43] Tordai H , Torres O, Csepi M et al Analysis of AlphaMissense data in different protein groups and structural context. Sci Data 2024;11:495. 38744964 10.1038/s41597-024-03327-8 PMC11094042

[btaf305-B44] Vaser R , Adusumalli S, Leng SN et al SIFT missense predictions for genomes. Nat Protoc 2016;11:1–9. 26633127 10.1038/nprot.2015.123

[btaf305-B45] Wu Y , Li R, Sun S et al Improved pathogenicity prediction for rare human missense variants. Am J Hum Genet 2021;108:1891–906. 34551312 10.1016/j.ajhg.2021.08.012 PMC8546039

[btaf305-B46] Zhang H , Xu MS, Fan X et al Predicting functional effect of missense variants using graph attention neural networks. Nat Mach Intell 2022;4:1017–28. 37484202 10.1038/s42256-022-00561-w PMC10361701

